# Chronic kidney disease, female infertility, and medically assisted reproduction: a best practice position statement by the Kidney and Pregnancy Group of the Italian Society of Nephrology

**DOI:** 10.1007/s40620-023-01670-4

**Published:** 2023-06-24

**Authors:** Rossella Attini, Gianfranca Cabiddu, Francesca Ciabatti, Benedetta Montersino, Andrea Roberto Carosso, Giuseppe Gernone, Linda Gammaro, Gabriella Moroni, Massimo Torreggiani, Bianca Masturzo, Domenico Santoro, Alberto Revelli, Giorgina Barbara Piccoli, On behalf of the Italian Society of Nephrology’s Project Group on Kidney and Pregnancy

**Affiliations:** 1 Department of Obstetrics and Gynecology SC2U, Sant’Anna Hospital, Città della Salute e della Scienza, Turin, Italy; 2 Nephrology, Department of Medical Science and Public Health, San Michele Hospital, G. Brotzu, University of Cagliari, Cagliari, Italy; 3 UOSVD di Nefrologia e Dialisi ASL Bari. P.O. “S. Maria degli Angeli”, Putignano, Italy; 4 Nephrology, Ospedale Fracastoro San Bonifacio, San Bonifacio, Italy; 5 Nephrology and Dialysis Division, IRCCS Humanitas Research Hospital, Rozzano, Milan, Italy; 6 Néphrologie et Dialyse, Centre Hospitalier Le Mans, 194 Avenue Rubillard, 72037 Le Mans, France; 7 Division of Obstetrics and Gynaecology, Department of Maternal-Neonatal and Infant Health, Ospedale Degli Infermi, University of Turin, Biella, Italy; 8 Unit of Nephrology and Dialysis, Department of Clinical and Experimental Medicine, A.O.U. “G. Martino”, University of Messina, 98125 Messina, Italy

**Keywords:** Chronic kidney disease, Medically assisted reproduction, Dialysis, Kidney transplantation, Infertility, Controlled ovarian stimulation, In vitro fertilisation, Ovarian hyperstimulation syndrome, Egg donation, Oocyte cryopreservation

## Abstract

Fertility is known to be impaired more frequently in patients with chronic kidney disease than in the general population. A significant proportion of chronic kidney disease patients may therefore need Medically Assisted Reproduction. The paucity of information about medically assisted reproduction for chronic kidney disease patients complicates counselling for both nephrologists and gynaecologists, specifically for patients with advanced chronic kidney disease and those on dialysis or with a transplanted kidney. It is in this context that the Project Group on Kidney and Pregnancy of the Italian Society of Nephrology has drawn up these best practice guidelines, merging a literature review, nephrology expertise and the experience of obstetricians and gynaecologists involved in medically assisted reproduction. Although all medically assisted reproduction techniques can be used for chronic kidney disease patients, caution is warranted. Inducing a twin pregnancy should be avoided; the risk of bleeding, thrombosis and infection should be considered, especially in some categories of patients. In most cases, controlled ovarian stimulation is needed to obtain an adequate number of oocytes for medically assisted reproduction. Women with chronic kidney disease are at high risk of kidney damage in case of severe ovarian hyperstimulation syndrome, and great caution should be exercised so that it is avoided. The higher risks associated with the hypertensive disorders of pregnancy, and the consequent risk of chronic kidney disease progression, should likewise be considered if egg donation is chosen. Oocyte cryopreservation should be considered for patients with autoimmune diseases who need cytotoxic treatment. In summary, medically assisted reproduction is an option for chronic kidney disease patients, but the study group strongly advises extensive personalised counselling with a multidisciplinary healthcare team and close monitoring during the chosen medically assisted reproduction procedure and throughout the subsequent pregnancy.

**Graphical abstract:**

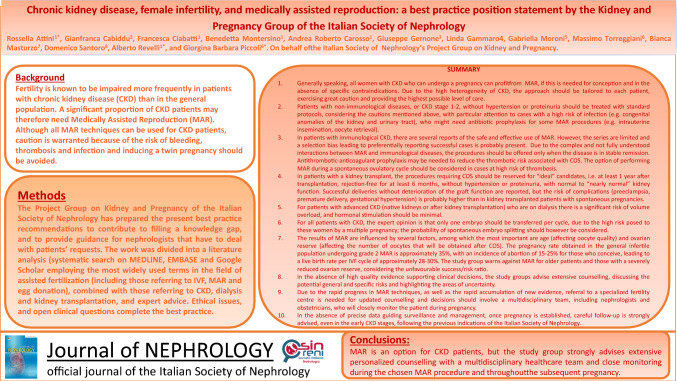

## Background

Infertility is defined as “the failure to establish a clinical pregnancy after 12 months of regular and unprotected sexual intercourse” [1]. It is a complex disorder with psychological, social and economic consequences. Ten years ago, the World Health Organization (WHO) estimated that 48.5 million couples worldwide were affected by infertility, and their number is constantly increasing [2].

Overall, patients of both sexes with chronic kidney disease (CKD) have lower fertility than the general population [3, 4]. In women with CKD, the causes of reduced fertility are only partially understood, but are likely to include the combination of endocrine abnormalities leading to ovulation defects, alterations in endometrial morphology, reduced libido and precocious consumption of the ovarian oocyte reserve [3, 5]. The ovarian reserve, estimated by measuring anti-Mullerian hormone (AMH) circulating levels, plays a crucial role in determining the duration and age limit of spontaneous fertility, as well as the efficacy of fertility-promoting treatments: patients with CKD stage 3–5 have lower AMH levels than the general population, although AMH levels in patients on haemodialysis may be high due to impaired glomerular filtration, limiting urinary clearance of the hormone [6].

There is no indication suggesting that proteinuria and hypertension, which, together with the degree of kidney function impairment, characterise CKD, are associated per se with a reduction of fertility, although some immunosuppressive treatments may reduce the ovarian reserve, as will be further discussed.

Medically Assisted Reproduction (MAR) is a definition applied to an array of treatments aimed at treating infertility, including in vitro fertilisation (IVF) [7]. While this term is presently preferred in the obstetrical setting, other synonyms are commonly used, including “assisted reproduction” or “assisted reproductive technologies” (ART). All these terms were used in our bibliographic searches [8].

A significant proportion of women affected by CKD may need MAR to resolve infertility problems. Unfortunately, the prevalence of CKD patients using MAR is not known; likewise, its potentially undesired effects, specifically in CKD patients, are only partially known (Table 1).

**Table 1:** Terminology and definitions [8]

MAR	Medically assisted reproduction (MAR)	Techniques that aim to help women with infertility problems become pregnant, in which lab procedures are involved
IVF-ET	In vitro fertilisation and embryo transfer (IVF-ET)	A MAR technique in which multiple ovulation is hormonally stimulated, oocytes are retrieved by ovarian puncture and then fertilized in vitro by co-incubation with prepared semen. The embryos obtained are cultured until the blastocyst stage, after which one blastocyst is implanted in the uterus
ICSI	Intra-cytoplasmic sperm injection (ICSI)	Similar to IVF-ET, but in vitro fertilisation is obtained by injecting a spermatozoon into each oocyte
COS	Controlled ovarian stimulation (COS)	Hormonal treatment aimed at inducing multiple follicle development in a controlled way, balancing efficacy and safety
OHSS	Ovarian hyperstimulation syndrome (OHSS)	Iatrogenic, excessive enlargement of the ovaries after COS, with liquid loss from the ovarian surface and subsequent ascites, thrombophilic conditions, electrolyte imbalance and abdominal pain. In its severe form, it is the most dangerous complication of MAR

## About these best-practices

The Project Group on Kidney and Pregnancy of the Italian Society of Nephrology has prepared the present best practice recommendations to contribute to fill a knowledge gap, and to provide guidance for nephrologists when dealing with patients’ requests. Our objective is to support nephrologists and gynaecologists in counselling CKD patients with infertility problems. There have been few studies on using MAR for CKD patients and the present recommendations collect and sort the scant data we have into two parts: the first deals with indications, the second with MAR techniques, highlighting the potential problems related to their application in different subsets of women with CKD.

The work was divided into a literature analysis (systematic search on MEDLINE, EMBASE and Google Scholar) employing the most widely used terms in the field of assisted fertilisation (including those referring to IVF, MAR and egg donation), combined with those referring to CKD, dialysis and kidney transplantation, and expert advice. Ethical issues, and open clinical questions complete the best practice.

Due to the lack of randomised controlled trials and of large observational studies, the level of evidence is relatively low for all statements (level 3–5, in which most of the level 3 evidence concerns MAR in non-CKD patients), and due to its homogeneity, the grade is not further reported in detail for each statement/recommendation.

The draft was circulated to the members of two groups of the Italian Society of Nephrology; one focused on kidney and pregnancy and another dealt with conservative treatment of CKD. All members were contacted by email and comments and answers were gathered by the steering committee.

Consensus was reached for all items listed in the “clinical advice” boxes:

- 1. General indications for counselling;
- 2. Overall advice on MAR techniques for CKD patients;
- 3. Measures to avoid severe ovarian hyperstimulation syndrome (OHSS) in CKD patients;
- 4. Elements to consider when discussing egg donation with CKD patients.

The Italian Best Practices, by policy, do not require patient involvement in the discussion. However, acknowledging that patient involvement is of the utmost importance, and is becoming an integrative part of every recommendation from scientific societies, the publication of the current paper will be followed by a patient’s commentary and by a lay summary, respectively published with free access and made available on the society website.

This best practice reflects the current obstetric nephrology management in a European setting; most of the retrieved papers were from high-income countries. However, we hope that the recommendations may be adapted to different settings, and increase interest while helping gather further, greatly needed evidence.

As a final note, infertility and a reduction in libido are also observed in males with CKD. Our decision to focus on women was motivated by the huge impact that both MAR and the subsequent pregnancy have on a woman’s health.

## Part one: infertility in women affected by CKD

### Chronic kidney disease

We have scant data on spontaneous fertility in CKD patients and there are several barriers to overcome to fill this important knowledge gap.

First, the majority of CKD patients are asymptomatic or oligosymptomatic until late in their clinical course. For example, IgA nephropathy may progress without episodes of macrohematuria; minor renal malformations and even reflux nephropathy are sometimes diagnosed only in adulthood; interstitial and cystic diseases may be completely asymptomatic, in particular in their early stages [9].

Second, there are few screening programs for CKD in young people. Even in pregnancy, urinalysis is the only test regularly advised in obstetrical guidelines, mainly for disclosure of proteinuria, since transient haematuria is observed in up to 20% of pregnancies [10]. The Italian Society of Nephrology holds that creatinine assessment should be added to the tests routinely performed in pregnancy, but this is still not being done [11]. Kidney ultrasounds or other imaging techniques are needed for the early diagnosis of many forms of CKD encountered in women of childbearing age (such as congenital anomalies of the kidney and urinary tract, and cystic diseases of the kidney). Although it is now acknowledged that CKD is associated with an increased risk of adverse pregnancy outcomes, our knowledge is limited to those with complications, which are probably the tip of the iceberg of CKD in women of childbearing age [12, 13].

As a consequence, there are no data on the prevalence of infertility in early CKD. While patients with advanced kidney disease are more likely to have received a CKD diagnosis, it is hypothesised that at least 50% of patients with advanced CKD are unaware that they have a kidney disease [11]. Notwithstanding these limitations, we have indirect data on some categories of patients with early CKD, namely those with primary glomerulonephritis and those with rheumatological diseases, of which systemic lupus erythematosus is the most prevalent [14].

It is generally believed that fertility decreases in parallel with the development of a series of hormonal derangements, whose mutual interactions are only partially known. The three main  explanations for infertility, and clues for restoring fertility are an increase in either prolactin levels or parathyroid hormone, and a decrease in erythropoietin, but other factors, such as vitamin deficiency or concomitant treatments, may play an important role. The importance of social context will be discussed below.

Since hormonal derangements of this sort usually become clinically evident only in stage 3b-5 CKD, when the glomerular filtration rate drops below 45 ml/min, it is generally thought that the decrease in fertility is proportional to the impairment of kidney function. This is likely to be true, but its extent is still unknown, and pregnancy is indeed possible at all CKD stages, and in patients on dialysis.

### Dialysis

Patients on renal replacement therapy have low fertility rates, and low chances of delivering a live-born baby. The figure often cited, which is derived from Australian and Italian data, reports a ratio of about 1: 10: 100 of having a pregnancy beyond 20 gestational weeks being on dialysis, having received a kidney transplantation, and being a part of the general population without known CKD, respectively [15, 16]. These data were recently challenged by a study conducted in the U.S., which reported a much higher conception rate for patients on dialysis (almost in the range of the kidney graft population), but this higher than expected fecundity was associated with only about 30% of live births, with an astonishingly high number of pregnancies with an unknown outcome (about 30%) [17]. In this context, pregnancy was more common in women from disadvantaged milieus and ethnic minorities, highlighting the importance of social context [17].

Table 2 reports the main risks to consider when a woman on dialysis wants to become pregnant, and some of the potential interventions. Many of them, including correction of anaemia, optimisation of the calcium-phosphate balance, careful nutritional management and, when necessary, correction of high prolactin levels, should be a routine part of good clinical practice in the management of young patients on dialysis. Data from Canada suggest that increasing dialysis dose for patients who want to become pregnant, for example by administering the same daily long-hour haemodialysis sessions employed during pregnancy, may increase the chances of conception, once again underlining how greater clearance of uraemic toxins may play a key role [18].

**Table 2:** Possible causes of infertility in dialysis patients, and potential interventions

Potential cause	Suggested treatments
Disturbed hormonal milieu	Increase dialysis frequency and efficiency up to daily long-hour dialysis
Hyperprolactinaemia	If increasing dialysis efficiency is not effective, cabergoline can be employed
Anaemia	Increase dialysis efficiency; optimise iron and erythropoietin therapy
Reduced libido	Assess psychological aspects; revise pharmacology
Malnutrition	Revise diet; liberalise diet and increase dialysis frequency; increase physical activity
Undiagnosed	Low fertility is most often multifactorial. Consider a trial of intensive dialysis, reduce drug burden and liberalise diet

### Kidney transplantation

It is widely believed that transplantation restores fertility in CKD women. However, fertility is only partially restored by kidney transplantation. Once more, available data are incomplete. Our knowledge mostly comes from the Australian and New Zealand Dialysis and Transplant Registry, the only one that has systematically gathered data on pregnancy in women who have undergone a transplant or been on dialysis, and from scattered sources, like the previously cited Italian nationwide survey, and voluntary registries. These are of value in that they establish that a risk of complications exists, but do little to enable us to more accurately calculate the fecundity rate [15, 18, 19].

Furthermore, since a patient with a kidney transplant is usually counselled to become pregnant only in the presence of healthy and stable kidney function, normal blood pressure, absent or low-grade proteinuria, and no recent rejection episode, data are not always easy to interpret, as they probably deal with the “best” subset of kidney transplant recipients, whose prevalence is not specified in many studies [20]. In addition, as in the case of dialysis, early miscarriages and voluntary pregnancy terminations are seldom recorded, suggesting a systematic underestimation of conception rates. Furthermore, drug choices may modulate advice: mycophenolate is one of the most effective drugs in kidney transplantation, but is associated with malformations and the policy towards discontinuation—treatment shift may indirectly affect pregnancy choices [21, 22].

### Social issues and notes on counselling

As previously mentioned, the geographic and ethnic distribution of pregnancies among dialysis patients indicates that social issues are a relevant factor. Pregnancies are more often reported in settings in which the cultural importance of the family is higher, and a kidney disease is less likely to be seen as an impairment to leading a normal life, due in some measure to its not being clearly “visible” [17, 23].

Only a few decades ago, in western countries, well-reviewed papers in prestigious journals, such as *The Lancet*, boldly stated that women with CKD should not conceive [24]. This negative attitude may have contributed to the low pregnancy rate in women with known CKD, and may at least partially explain the paradox of a higher conception rate in underserved minorities [17].

Reproductive autonomy is an increasingly acknowledged issue; merging its multiple dimensions may be difficult in CKD, but should be our goal [25–27].

Counselling on pregnancy in CKD is difficult and requires sensitivity. It is unlikely that the same policy will be chosen in all settings, as, even in Europe, the priorities given to different ethical principles may vary. For example in Mediterranean countries the principle of non-maleficence (“*primum non nocere*”) tends to be paramount, while in the U.K. and other parts of northern Europe, autonomy is generally considered more important [28–30].

The Project Group on Kidney and Pregnancy supports informative counselling with thorough discussion of the unresolved issues and unclear or only partial data, holding that the patient’s decision should be respected and supported, pursuing a patient-physician relationship of concordance and therapeutic alliance.

Box 1: General indications for counselling **What a woman with CKD who wants to become pregnant needs to know**

Pregnancy is possible in all CKD stages

Fertility is thought to be low in the late CKD stages and is known to be low in dialysis and transplanted patients

Fertility is only partially restored by kidney transplantation

Pregnancy in CKD is not associated with an increase in malformations

In high-income countries, pregnancy in CKD is not associated with an increased risk of maternal death

Medically Assisted Reproduction techniques are feasible in all CKD stages

**What are the risks (during and after pregnancy) for a woman with CKD who wants to become pregnant**

CKD is associated with an increased risk of adverse pregnancy outcomes in all CKD stages

Pregnancy in advanced CKD or with a failing kidney graft is associated with a more rapid progression of kidney disease

If pregnancy complications develop, the progression of CKD may be more rapid

Medically Assisted Reproduction increases the risk of adverse pregnancy outcomes, and this increases from grade 1 to grade 2 techniques to egg donation

Combining CKD and Medically Assisted Reproduction is likely to further increase the risk of adverse pregnancy-related outcomes; the degree of this risk is for the moment unknown

Patients on potentially teratogenic drugs (non-specific for CKD) carry specific risks if these drugs are not promptly discontinued

Patients with hereditary kidney diseases carry the risk linked to the type of genetic inheritance of their disease

Children born prematurely, with low birth weight or with intrauterine growth restrictions are probably more prone to metabolic syndrome, hypertension and CKD in adulthood

## Part two: medically assisted reproduction for patients with CKD

### Medically assisted reproduction (MAR) techniques

Medically Assisted Reproduction includes grade 1 techniques, in which fertilisation occurs inside the woman’s body, and grade 2 techniques, in which the oocyte is fertilised outside the woman’s body, in a laboratory setting [31].

Grade 1 MAR includes all forms of insemination, using either sperm from the woman’s partner or from a donor. By far, the most frequently used technique is intra-uterine insemination (IUI), in which an aliquot of semen processed in the laboratory is gently injected inside the uterus at the time of ovulation [32]. Other forms of insemination (e.g. intracervical, intraperitoneal) have now been almost entirely abandoned.

**Fig. 1:**
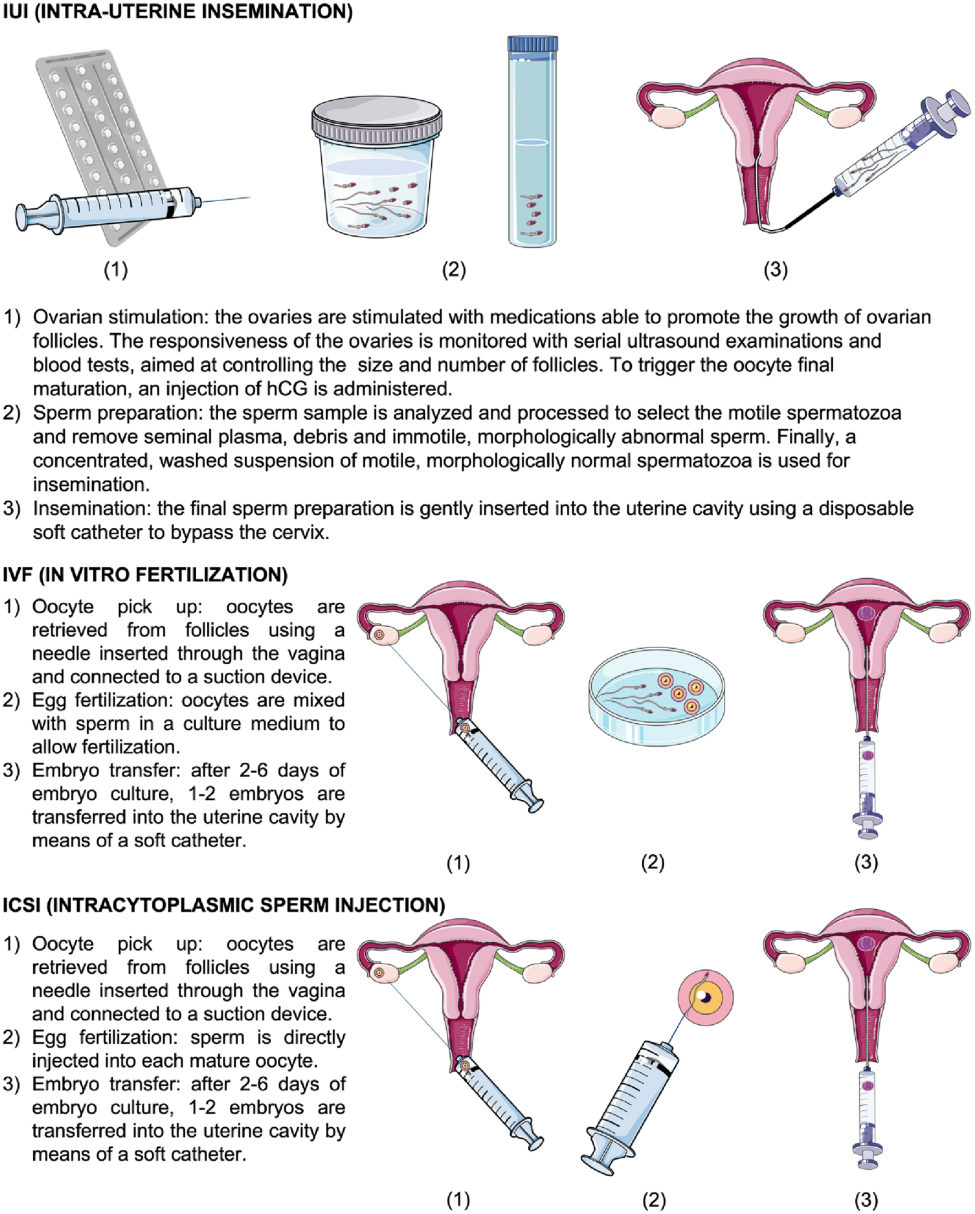
Schematic description of the different MAR techniques

The most widely-used Grade 2 MAR techniques are in vitro fertilisation and embryo transfer (IVF-ET). Another technique is intra-cytoplasmic sperm injection (ICSI), a variant of IVF-ET, in which spermatozoa are directly injected inside the oocytes in order to increase the chance of fertilisation, even when the semen quality is poor (Fig. 1). These two basic techniques are sometimes implemented applying embryo biopsy with pre-implantation genetic testing for aneuploidy (PGT-A) or for specific mutations (PGT-M) or translocations (PGT-T) associated with genetic diseases [33]. The option of preimplantation embryo selection should be discussed with patients affected by a hereditary kidney disease, including Alport syndrome and autosomal dominant polycystic kidney disease (ADPKD). The ethical issues arising when choosing between a spontaneous pregnancy at risk for a chronic disease and a pregnancy needing complex fertilisation techniques should be discussed case by case [34–36].

Embryo cryopreservation is also frequently used, both to prevent complications, like OHSS or multiple pregnancies, and to increase success rates by transferring extra embryos in subsequent ovarian cycles [37]. Both IVF-ET and ICSI can be performed using donor gametes, if needed.

#### Hormonal ovarian stimulation

The early steps in hormonally-stimulated ovulation are the same in grade 1 and grade 2 MAR techniques, with the difference being that in grade 1 MAR, when conception occurs inside the woman’s body, monofollicular ovulation will occur, whereas when IVF-ET or ICSI are performed, multifollicular ovulation is needed to optimise the chances of success. The dose of exogenous gonadotropins (Gn), administered to stimulate the ovary, changes according to the aim of stimulation (one or several growing follicles), and the risk of undesired effects are directly proportional to the dose the patient receives.

Hormonal ovarian stimulation is accomplished by prescribing subcutaneously-injectable medications for approximately 2 weeks. They are usually self-administered and contain either follicle-stimulating hormone (FSH), luteinising hormone (LH) or a 1:1 combination of both (hMG). Serial vaginal ultrasound examinations are performed to monitor the development of ovarian follicles [38]. In grade 2 MAR, spontaneous follicular rupture, which would compromise the possibility of retrieving oocytes, is prevented by blocking the pituitary gland, using Gonadotropin-releasing hormone (GnRH) antagonists or agonists, to avoid an endogenous LH peak. This complete pharmacological control over the ovulation regulating system accounts for the name given to the whole hormonal treatment: Controlled Ovarian Stimulation (COS).

#### Oocyte retrieval

To maximise the chances of retrieving mature oocytes (follicular diameter between 16 and 23 mm), COS is continued until the target follicular development is reached. Subsequently, LH surge is mimicked by administering a subcutaneous bolus of human chorionic gonadotropin (HCG), which triggers the final oocyte maturation and the resumption of meiosis in the female gamete. Within 34–36 h after HCG injection, oocytes are retrieved using transvaginal ultrasound-guided ovarian puncture, with follicular fluid aspiration (Fig. 1). This procedure is usually performed under loco-regional anaesthesia (paracervical block) and deep sedation, and only exceptionally is general anaesthesia required. Surgical complications such as haemorrhage, injury of the pelvic structures, and pelvic infection are rare [39, 40].

#### Fertilisation

The semen sample is obtained by masturbation on the morning of egg retrieval. Other methods (e.g. the use of a needle or surgical procedure to extract sperm directly from the testicle) may be required in the presence of extremely severe male infertility (cryptozoospermia) [41]. Donor sperm can be used if there is total absence of spermatozoa (azoospermia) even inside the testicle [42, 43].

Fertilisation in the laboratory can be obtained either by maintaining contact between in vitro-activated spermatozoa and mature eggs (conventional IVF-ET), or directly injecting a carefully selected spermatozoon (normal morphology and motility) in each oocyte (ICSI). Fertilised eggs (zygotes) are then cultured in artificial incubators with controlled atmospheres and specific media for five days, until embryo development reaches the blastocyst stage, when the zygotes can be transferred into the uterus. After embryo transfer, progesterone is administered at high doses (e.g. 600 mg/d as vaginal suppositories) in order to balance high estradiol levels and promote optimal endometrial maturation.

In recent years, the policy of transferring a single blastocyst has increased, mainly in high-income countries, with the aim of reducing the rate of twin and triplet pregnancies, as well as the risk of OHSS, the two most frequent complications of MAR treatments [44].

#### Pregnancy rate

Since 1997, MAR aggregated data generated by national registries, hospitals or professional societies have been gathered and analysed by the European IVF-monitoring Consortium (EIM). From the latest available data referring to 2017, 1382 hospitals offering ART services in 39 countries reported a total of 940,503 grade 2 treatment cycles; the pregnancy rate per embryo transfer in 2017 was 34.8%; twin and triplet delivery rates for grade 2 MAR cycles were 14.9% and 0.3%, respectively [45].

Thus, the overall effectiveness of MAR has remained constant, but the twin and triplet rates have been significantly reduced by avoiding the transfer of more than one blastocyst at a time, and by extensively employing cryopreservation for unused blastocysts.

### Controlled ovarian stimulation

Controlled ovarian stimulation with gonadotropins is an essential part of MAR treatments, as the correct use of COS reduces the risk of ovarian hyperstimulation syndrome.

#### COS in grade 1 MAR

In grade 1 MAR, COS is aimed at obtaining a single oocyte and synchronising ovulation with sperm injection in the uterine cavity.

Intra-uterine insemination can also be performed during spontaneous ovulation, without using exogenous hormones, but the results are inferior to those obtained when gonadotropins are administered [46]; it is likely that this is due to the relatively short survival of in vitro-activated spermatozoa and to the difficulty of precisely predicting the time of spontaneous ovulation, with problems in synchronising sperm survival and egg availability. When a low dose of gonadotropins is used, their circulating levels are close to those observed in a spontaneous cycle, and therefore complications are extremely rare, and the risk of the most challenging of them, severe OHSS, is considered negligible.

#### COS in grade 2 MAR

In grade 2 MAR, the ultimate aim of COS is to obtain the number of oocytes that will maximise the success rate, while keeping the risk of OHSS under control: based on the current evidence, the optimal number of oocytes should range between 15 and 18 [47]. The simultaneous optimisation of COS efficacy and safety can be achieved by tailoring treatment according to biomarkers that predict ovarian response to COS. Algorithms have been developed to calculate the optimal dose of exogenous gonadotropins that should be administered to a given woman [48, 49]; they combine clinical characteristics (e.g. age and body mass index [BMI]) with ultrasound parameters (basal antral follicle count [AFC]) and serological markers (FSH level on the third day of the cycle, circulating anti-Mullerian hormone level) [48–50]. Briefly, the antral follicle count is the number of small antral follicles (2–10 mm diameter) that are visible at transvaginal ultrasound in both ovaries; it is directly related to ovarian responsiveness to COS [51]. The anti-Mullerian hormone, which is produced by the granulosa cells of pre-antral follicles, is the individual biomarker that most closely correlates with the follicular ovarian reserve and response to COS; conversely, FSH secretion is inhibited by inhibin B, another product of the granulosa cells, and FSH levels on the third day of the cycle are inversely related to the size of the woman’s ovarian reserve [52].

#### Drugs employed in COS

The drugs used for COS are human gonadotropins FSH, LH and HCG; they are obtained by extraction and purification from the urine (u-Gn) or using recombinant technology (r-Gn) [53]. Both u-Gn and r-Gn are used in COS, with small differences in efficacy and safety. Most COS cycles are performed using either FSH alone, or a combination of FSH plus LH or FSH plus HCG (acting on the LH-receptor and having LH-like activity). Several Gn administration regimens exist and this makes it possible to choose the one that is most likely to ensure efficacy and safety for a particular patient [52]. Overall, COS lasts approximately two weeks and the largest administered Gn dose very rarely exceeds 300–375 IU/day, reaching a maximum dose of 5000 IU per stimulation cycle.

The ovarian response to COS consists in the simultaneous growth of a cohort of follicles which then produces estradiol. During COS, estradiol circulating levels are ten times higher than those of the pre-ovulatory peak in a spontaneous cycle (about 3000 pg/ml vs. about 300). Most gonadotropins are administered daily by subcutaneous injection to reach a steady and active blood concentration within the fourth day of treatment; it takes approximately three days for changes in daily dose to be reflected in the blood concentration [53].

The pharmacokinetics described above applies to women with normal kidney function, as gonadotropins are cleared via urinary excretion; the pharmacodynamic pattern changes in cases of impaired kidney function, potentially leading to Gn accumulation and the need to modulate the dose. However, there is a well-known limit in the responsiveness of granulosa cells to Gn, due to Gn-receptor saturation, which prevents any further effect on the follicles.

A long-acting form of recombinant FSH (r-FSH), Corifollitropin-alfa (CF-alfa), obtained by coupling the r-FSH molecule with the C-terminal sequence of HCG, is also available; thanks to the added peptide fragment, r-FSH has a four times longer half-life in the blood [54]. A single CF-alfa injection takes only two days to reach active concentration in the blood, and leads to seven days of steady circulating FSH concentrations. The concentration of these long-lasting products could significantly increase in advanced CKD.

### Advice on using COS for women with CKD

When treating CKD patients, low-dose COS should be preferred, since the risks associated with severe OHSS (ascites, hypovolemia, hypoproteinemia, electrolyte imbalance and thrombophilia) increase in the presence of CKD, in particular in oedematous states or in advanced CKD stages [55, 56].

The precise pharmacodynamics of stimulating hormones in CKD is not known; overall, long-lasting stimulating hormones should be used very carefully for patients with kidney impairment, given the increased risk of excessive FSH stimulation linked to reduced kidney clearance.

Given the oestrogen-related risk of flares, particular care should be taken when treating patients with lupus nephritis [57, 58]. In these cases, drugs that control oestrogen rise, such as the aromatase inhibitor Letrozole or the oestrogen receptor antagonist Clomiphene citrate, can effectively and safely be associated with gonadotropins [59]. In given circumstances, even grade 2 MAR can be successful when a single oocyte is retrieved during a spontaneous, unstimulated cycle, accepting a much lower efficacy of the cycle, but significantly reducing the risk of disease reactivation thanks to the reduced risk of a Gn-related oestrogen rise [60].

For patients affected by SLE, pregnancy rates after grade 2 MAR are similar to those for the general population, but flares, OHSS, spontaneous abortion, preeclampsia and preterm delivery seem to be more frequent, according to some case series, most of which were based on relatively small samples [61–65].

The association between CKD and multiple pregnancies is known to carry an increased rate of adverse outcomes compared to multiple pregnancies in individuals without CKD; inducing a multiple pregnancy in CKD should therefore be avoided [66].

Patients should be warned that even when a single blastocyst is transferred, a twin pregnancy (monochorionic twins) can be caused by spontaneous embryo splitting; however, the risk is low, i.e. below 4% [67].

Even if the risk of bleeding after oocyte retrieval is low, it should be kept in mind, in particular for patients on haemodialysis, as they use intermittent heparin during dialysis, and the same caution exercised for a surgical procedure should be applied here [68].

Similarly, even if the risk of infection related to this procedure is low, pre-procedure vaginal bacteriological examination and/or antibiotic prophylaxis should be considered, at least for patients on immunesuppressive drugs and patients at high risk of pelvic infection***.***

As a note of caution, it should be mentioned that the high oestrogen levels produced when using a supra-physiological concentration of circulating progesterone may increase a patient’s thrombophilic status, and the thrombotic risk may be further accentuated by a sedentary life in the days following embryo transfer. These factors should be considered in women at high risk for thrombosis, for example those with nephrotic syndrome or advanced CKD, obesity, a history of recurrent thrombosis or the presence of anti-phospholipid antibodies, also outside the context of a full-blown antiphospholipid syndrome. For these patients, antithrombotic prophylaxis with low molecular weight heparin should be considered, whenever possible combined with low-dose aspirin, depending on individual risks. Low-dose aspirin should be stopped three days before oocyte retrieval and resumed the day after; low molecular weight heparin should be stopped 12 h before retrieval and may be resumed the same day under strict control [58].

Box 2. Overall advice on MAR techniques for CKD patients In the absence of agreed indications for monitoring CKD patients during and after MAR, the study group holds that patients should be advised to:

- **Avoid a multiple pregnancy** *(indirect evidence stemming from data on multiple pregnancies in CKD patients)*
- **Check weight and blood pressure daily, and report any rapid increase in weight and any increase in blood pressure** *(good clinical practice in high-risk pregnancies)*
- **Use prophylactic aspirin or heparin at the prescribed distance from oocyte pick-up, according to the baseline situation (especially sedentary patients and patients with other thrombotic risk factors)** *(indirect evidence from data on specific subsets of CKD patients, and on large series of MAR treatments)*
- **Avoid intradialytic heparin in haemodialysis at least 12 h before and 24 h after the oocyte retrieval procedure** *(evidence from case reports)*
- **Choose cautious, low-dose ovarian stimulation** *(indirect evidence from MAR treatments and clinical reasoning; for details, see Box 3, below)*

### Prevention of severe ovarian hyperstimulation syndrome

Ovarian hyperstimulation syndrome is an iatrogenic complication that can affect patients undergoing COS; in its severe form it is the only life-threatening complication of COS [69].

In the general population of women undergoing COS, severe OHSS is rare, affecting 0.1–0.2% of patients [70], but in specific subsets (e.g. young women with polycystic ovary syndrome) its prevalence may be as high as 6%. The milder forms of OHSS are more frequent and are often treated on an outpatient basis, without need for hospitalisation.

The risk of developing severe OHSS increases when an excessive ovarian response to COS occurs as a consequence of an overly-high dose of exogenous gonadotropins or of unpredicted, excessive ovarian sensitivity to gonadotropins, linked to a multifactorial genetic predisposition. The intense ovarian response to COS leads to an abrupt and very marked increase in circulating estradiol, stimulating, via the vascular endothelial growth factor (VEGF), the vascular permeability of the small vessels on the ovarian surface. The administration of HCG to trigger ovulation is the final factor activating VEGF, leading to fluid outflow from the ovarian surface into the abdominal cavity [71].

The full clinical picture of severe OHSS usually appears five to ten days after HCG has been administered, and includes enlarged ovaries (up to 12 cm in diameter), ascites, hypovolemia, hypoproteinemia, electrolyte imbalance and thrombophilic status (Table 3). In the most severe cases, pleural effusion with respiratory distress may occur; hypovolemia-linked hypoperfusion of the vital organs (including the kidneys) may lead to damage, and even death [69].

**Table 3:** Aboulghar and Mansour’s classification of OHSS by level of risk [72]

Grade	Symptoms and signs	
Moderate	Abdominal tension, nausea, ovarian enlargement, ascites, normal blood tests	
Severe Grade A	Nausea and/or vomiting, oliguria, ovarian enlargement, ascites, slight dyspnoea, normal blood tests	
Severe Grade B	Abdominal pain, nausea and/or vomiting, severe oliguria, ovarian enlargement, ascites, severe dyspnoea, haemoconcentration, high liver enzymes, electrolyte disturbances	
Severe Grade C	Same symptoms as in grade B, but associated with at least one of the following: Acute Respiratory Distress Syndrome (ARDS), thromboembolic events, acute kidney injury (AKI)	

The treatment of severe OHSS consists of intravenous fluids and albumin, anticoagulant low molecular weight heparin prophylaxis, electrolyte balancing and cabergoline, a drug inhibiting class II VEGF receptor [69].

#### Advice on the prevention of OHSS in CKD

The incidence of severe OHSS in patients with CKD is not known, but some case reports have been published [73–76]. Patients with CKD should be considered at high risk of further kidney damage in case of severe OHSS, and great caution to avoid the syndrome should be exercised. It is in fact vital that every measure that can be adopted to minimise the risk of severe OHSS [77] should be employed in treating patients with CKD (Box 3).

Box 3: Measures to avoid severe ovarian hyperstimulation syndrome in CKD patients **Suggested measures for avoiding severe OHSS**

Using a COS protocol with a 30% lower-than-normal gonadotropin dose coupled with the administration of a GnRH-antagonist to inhibit the pituitary gland; this allows us to replace HCG with a GnRH-agonist to trigger final follicular maturation, thus avoiding the HCG effect on VEGF

Associating a prophylactic dose of cabergoline with COS to control the VEGF effect and minimise the increase in vascular permeability

Discontinuing COS if even minor signs of OHSS occur, e.g. liquid leakage from the ovaries (visible as free-fluid at transvaginal ultrasound); an abrupt and excessive increase in circulating estradiol

Avoiding embryo transfer during the cycle in which COS has been performed. Embryos can be frozen and transferred during the following menstrual cycle, either after preparing the endometrium with oestrogen plus progesterone, or during a spontaneous luteal phase. This prevents starting a pregnancy with endogenous HCG secretion by the placenta

### Oocyte donation

Women with premature ovarian insufficiency or who are over 40–43 years of age (the age limit varies from country to country), as well as those who have a very limited ovarian reserve or no longer have good quality oocytes as a consequence of ageing, can still become pregnant through oocyte donation from a healthy young woman. Oocyte donors are younger than 35 and have been tested for genetic, metabolic, psychiatric and infectious diseases. One embryo generated in vitro from a donor oocyte and the patient’s partner’s sperm is transferred into the patient’s uterus, with a 50–60% chance of starting a pregnancy and a slightly lower live birth rate (45–55%).

Probably due to alterations in immune-tolerance toward the embryo, which is genetically different from the recipient, hypertensive disorders and foetal growth restriction are significantly more frequent after oocyte donation than after homologous IVF or in a spontaneous pregnancy [78].

### Advice on egg donation in CKD

Specifically when treating CKD women, it should be kept in mind that egg donation increases the risk of the hypertensive disorders of pregnancy, which are in turn associated with a higher risk of CKD progression, even in the early CKD stages. These considerations should be discussed with patients at the time of their selection of a MAR procedure.

Box 4. Elements to consider when discussing egg donation with CKD patients **Pro:** relatively high success rate (about 50% of pregnancies last beyond the first trimester)

**Contra**: increased risk of the hypertensive disorders of pregnancy ( the incidence of which is already increased in all stages of CKD), which are in turn associated with a higher risk of CKD progression

## Part three: preserving fertility in patients with CKD

Fertility preservation applies to women under 40, since after this age oocyte quality is considered to be too poor to warrant any attempt to preserve them. This procedure should be considered for all patients that have a higher than normal risk of losing significant amounts of their ovarian follicular reserve because of severe multisystem autoimmune diseases or therapies that are potentially toxic for the ovaries [79].

The EULAR guidelines warn that methotrexate, mycophenolate mofetil and cyclophosphamide are ovarotoxic medications, and suggest considering employing fertility preservation techniques before treatment [80].

The first option in preserving female fertility is to administer GnRH-analogues or oral contraceptives before ovarotoxic therapy is begun [81, 82]. The rationale is to block follicular growth and ovulation by inhibiting pituitary FSH and LH secretion; in addition, the pituitary block reduces the metabolic activity of the ovaries and their vascularisation, rendering follicles less vulnerable. The real efficacy of a GnRH-analogue or oral contraceptives in preserving the ovarian reserve of small follicles is controversial, as the early stages of follicular activation are independent from the stimulation of gonadotropins, and are due exclusively to intraovarian factors. However, some clinical evidence shows that these treatments have a beneficial effect, and they are still quite widely used in clinical practice [83].

The procedures currently being used to preserve female fertility are cryostorage of oocytes, embryos or ovarian tissue fragments [84].

Oocyte cryopreservation has been used since the 1990s and is a consolidated technique. The thousands of babies born using this procedure testify to its efficacy and safety. Cryopreservation is performed after COS and oocyte retrieval as in IVF treatment. After retrieval, the non-fertilised oocytes are frozen with a quick and effective method called vitrification, and are then stored in liquid nitrogen. They can survive frozen for several years and continue to produce competent embryos [85, 86]. When thawed, they can be fertilised using ICSI, without an increased risk of congenital anomalies [87].

The cryopreservation of embryos is likewise well known, tested, effective and safe, but the laws in some countries (e.g. Germany, Switzerland) restrict its use to married women, and in other countries (e.g. Italy) allow it only in specific cases that do not include fertility preservation for non-malignant diseases [88].

The cryopreservation of ovarian tissue is still experimental. It consists of a laparoscopic biopsy sampling of the ovarian cortex that is then frozen and stored in liquid nitrogen. This procedure is rapid, requiring three to four days vs. the 15 days needed for COS plus oocyte cryostorage, and could be offered to patients who need urgent treatment with cytotoxic drugs. After thawing, the ovarian tissue can be grafted by laparoscopy in the residual atrophic ovary. The efficacy of this procedure is not known: about 150 live-born babies are reported to date, and no assessment of the risk of foetal abnormality is possible [89, 90].

### Advice on using cryopreservation for women with CKD

Oocyte cryopreservation is the only widely available technique in clinical practice. Unlike other diseases and conditions, in case of glomerulonephritides the use of immunosuppressive treatments is generally of shorter duration. Since therapeutic alternatives to gametotoxic immunosuppressants exist both in chronic immunological kidney diseases and in kidney transplantation, the use of cryopreservation techniques is not systematically advised. It can, however be considered in selected cases.

A still open question is whether certain drugs, such as mycophenolate, need to be at least temporarily discontinued in women on treatment for the purpose of egg harvest for egg preservation.

Ideally, gamete retrieval should be performed before the start of treatment, in analogy with the indications for cancer therapy. However, this may not be feasible in some situations, such as for example, in patients with SLE, treated since adolescence, or in patients on chronic treatment who previously considered pregnancy. Pending future research, and acknowledging the complexity and the open discussion on this issue, the study group suggests case-by-case management with experts providing updated advice.

## Part 4: open clinical and ethical issues: optimising timing of pregnancy

One of the problems of fertility is the time issue. The chances of becoming pregnant and having an uncomplicated pregnancy decrease with age; the progression of kidney disease may be long. Therefore, determining the “right time” and making the “right decision” may be difficult, in particular in women nearing the need for renal replacement therapy or on dialysis.

While there is general agreement that conception is more frequent and a pregnancy has a better chance of being successful after kidney transplantation than on dialysis and, probably, with severe CKD, this has to be balanced against the fact that, even in the best circumstances, not every kidney transplant guarantees “ideal” kidney function. In the absence of a living donor, the waiting time may be long, and at least one rejection-free year after transplantation should elapse before pregnancy is undertaken [91, 92].

Although pregnancy in dialysis is feasible, and the results are improving, extended daily or nightly treatments are extremely demanding and not universally available; their results are in any case inferior to those reported after kidney transplantation [18].

Even if, overall, kidney transplantation results are improving, there is no certainty that normal or “nearly normal” kidney function will be attained after transplant, and the currently prescribed delay of at least one rejection-free year, with normal kidney function, no hypertension and non-relevant proteinuria before beginning a pregnancy is unlikely to change in the near future [93].

Older age is associated with an increased risk of chromosomal anomalies (aneuploidy), first of all Down syndrome. Although these can easily be diagnosed in utero, the psychological impact may be considerable. Furthermore, a pregnancy in advanced CKD carries a high risk of precipitating end-stage kidney disease and the need for dialysis [94, 95].

**Fig. 2:**
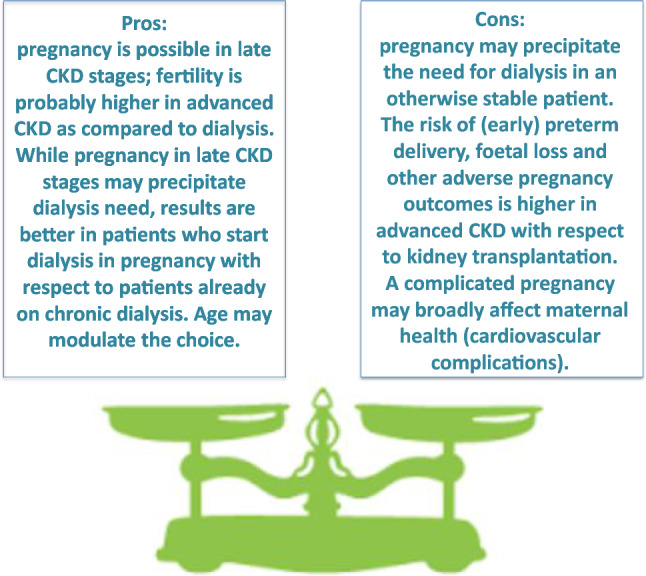
Timing and main clinical issues with regard to pregnancy planning in late CKD stages (3–5)

**Fig. 3:**
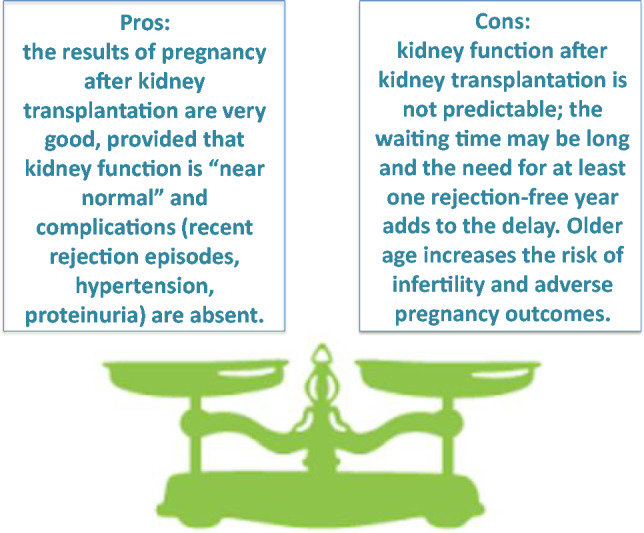
Timing and main clinical issues with regard to pregnancy planning after kidney transplantation

While cryopreservation of eggs is a feasible option, it does not completely solve the age problem, and the balance between an earlier pregnancy, in pre-dialysis or dialysis, versus a later one, possibly requiring assisted fertilisation, after kidney transplantation is not clear (Figs. 2, 3).

This review does not discuss the clinical issues linked to pregnancy management in patients with CKD, dialysis and transplantation, which are covered by past best practices [93, 96, 97]. Likewise, this paper does not discuss risk assessment, that should be a part of the counselling before MAR and is usually undertaken in these “high risk” situations; the importance of such a careful discussion is implicit and should, in any case, be underlined.

## Summary considerations

1. Generally speaking, all women with CKD who can undergo a pregnancy can profit from MAR, if this is needed for conception and in the absence of specific contraindications. Due to the high heterogeneity of CKD, the approach should be tailored to each patient, exercising great caution and providing the highest possible level of care (Boxes 1–4).
2. Patients with non-immunological diseases, or CKD stage 1–2, without hypertension or proteinuria should be treated with standard protocols, considering the cautions mentioned above, with particular attention to cases with a high risk of infection (e.g. congenital anomalies of the kidney and urinary tract), who might need antibiotic prophylaxis for some MAR procedures (e.g. intrauterine insemination, oocyte retrieval).
3. In patients with immunological CKD, there are several reports of the safe and effective use of MAR. However, the series are limited and a selection bias leading to preferentially reporting successful cases is probably present. Due to the complex and not fully understood interactions between MAR and immunological diseases, the procedures should be offered only when the disease is in stable remission. Antithrombotic-anticoagulant prophylaxis may be needed to reduce the thrombotic risk associated with COS. The option of performing MAR during a spontaneous ovulatory cycle should be considered in cases at high risk of thrombosis.
4. In patients with a kidney transplant, the procedures requiring COS should be reserved for “ideal” candidates, i.e. at least 1 year after transplantation, rejection-free for at least 6 months, without hypertension or proteinuria, with normal to “nearly normal” kidney function. Successful deliveries without deterioration of the graft function are reported, but the risk of complications (preeclampsia, premature delivery, gestational hypertension) is probably higher than in kidney transplanted patients with spontaneous pregnancies.
5. For patients with advanced CKD (native kidneys or after kidney transplantation) who are on dialysis there is a significant risk of volume overload, and hormonal stimulation should be minimal.
6. For all patients with CKD, the expert opinion is that only one embryo should be transferred per cycle, due to the high risk posed to these women by a multiple pregnancy; the probability of spontaneous embryo splitting should however be considered.
7. The results of MAR are influenced by several factors, among which the most important are age (affecting oocyte quality) and ovarian reserve (affecting the number of oocytes that will be obtained after COS). The pregnancy rate obtained in the general infertile population undergoing grade 2 MAR is approximately 35%, with an incidence of miscarriage of 15–25% for those who conceive, leading to a live birth rate per IVF cycle of approximately 28–30%. The study group warns against MAR for older patients and those with a severely reduced ovarian reserve, considering the unfavourable success/risk ratio.
8. In the absence of high quality evidence supporting clinical decisions, the study groups advise extensive counselling, discussing the potential general and specific risks and highlighting the areas of uncertainty (Box 1, Fig. 2).
9. Due to the rapid progress in MAR techniques, as well as the rapid accumulation of new evidence, referral to a specialised fertility centre is needed for updated counselling, and decisions should involve a multidisciplinary team, including nephrologists and obstetricians, who will closely monitor the patient during pregnancy.
10. In the absence of precise data guiding surveillance and management, once pregnancy is established, careful follow-up is strongly advised, even in the early CKD stages, following the previous indications of the Italian Society of Nephrology.

## Data Availability

Not available.
