# Correction to: Chronic kidney disease, female infertility, and medically assisted reproduction: a best practice position statement by the Kidney and Pregnancy Group of the Italian Society of Nephrology

**DOI:** 10.1007/s40620-024-01980-1

**Published:** 2024-05-09

**Authors:** Rossella Attini, Gianfranca Cabiddu, Francesca Ciabatti, Benedetta Montersino, Andrea Roberto Carosso, Giuseppe Gernone, Linda Gammaro, Gabriella Moroni, Massimo Torreggiani, Bianca Masturzo, Domenico Santoro, Alberto Revelli, Giorgina Barbara Piccoli, On behalf of the Italian Society of Nephrology’s Project Group on Kidney and Pregnancy

**Affiliations:** 1 Department of Obstetrics and Gynecology SC2U, Sant’Anna Hospital, Città della Salute e della Scienza, Turin, Italy; 2 Nephrology, Department of Medical Science and Public Health, San Michele Hospital, G. Brotzu, University of Cagliari, Cagliari, Italy; 3 UOSVD di Nefrologia e Dialisi ASL Bari. P.O. “S. Maria degli Angeli”, Putignano, Italy; 4 Nephrology, Ospedale Fracastoro San Bonifacio, San Bonifacio, Italy; 5 Nephrology and Dialysis Division, IRCCS Humanitas Research Hospital, Rozzano, Milan, Italy; 6 Néphrologie et Dialyse, Centre Hospitalier Le Mans, 194 Avenue Rubillard, 72037 Le Mans, France; 7 Division of Obstetrics and Gynaecology, Department of Maternal-Neonatal and Infant Health, Ospedale Degli Infermi, University of Turin, Biella, Italy; 8 Unit of Nephrology and Dialysis, Department of Clinical and Experimental Medicine, A.O.U. “G. Martino”, University of Messina, 98125 Messina, Italy

**Correction to: Journal of Nephrology (2023) 36:1239–1255** 10.1007/s40620-023-01670-4

The article “Chronic kidney disease, female infertility, and medically assisted reproduction: a best practice position statement by the Kidney and Pregnancy Group of the Italian Society of Nephrology”, written by Rossella Attini, Gianfranca Cabiddu, Francesca Ciabatti, Benedetta Montersino, Andrea Roberto Carosso, Giuseppe Gernone, Linda Gammaro, Gabriella Moroni, Massimo Torreggiani, Bianca Masturzo, Domenico Santoro, Alberto Revelli, Giorgina Barbara Piccoli, On behalf of the Italian Society of Nephrology’s Project Group on Kidney and Pregnancy, was originally published without open access. After publication in volume 36, issue 5, page 1239–1255 the author decided to opt for Open Choice and to make the article an Open Access publication. Therefore, the copyright of the article has been changed to © The Author(s) 2020 and the article is forthwith distributed under the terms of the Creative Commons Attribution 4.0 International License, which permits use, sharing, adaptation, distribution and reproduction in any medium or format, as long as you give appropriate credit to the original author(s) and the source, provide a link to the Creative Commons licence, and indicate if changes were made. The images or other third party material in this article are included in the article’s Creative Commons licence, unless indicated otherwise in a credit line to the material. If material is not included in the article’s Creative Commons licence and your intended use is not permitted by statutory regulation or exceeds the permitted use, you will need to obtain permission directly from the copyright holder. To view a copy of this licence, visit http://creativecommons.org/licenses/by/4.0.

The original article has been corrected.

